# Purpose in life and coping strategies: Main associations and moderation by concurrent distress

**DOI:** 10.1371/journal.pone.0347777

**Published:** 2026-05-21

**Authors:** Angelina R. Sutin, Martina Luchetti, Yannick Stephan, Amanda A. Miller, Antonio Terracciano

**Affiliations:** 1 Florida State University College of Medicine, Tallahassee, Florida, United States of America; 2 Euromov, University of Montpellier, Montpellier, France; University of Macerata: Universita degli Studi di Macerata, ITALY

## Abstract

Individuals with more purpose in life tend to report less subjective stress. This association may be due, in part, to the use of more adaptive coping strategies. This research examines how purpose is associated with common coping strategies and whether the relations differ by either sociodemographic factors or current psychological distress. Participants (*N* = 1,998) completed a survey that included purpose in life, coping strategies, current symptoms of depression and anxiety, and current feelings of stress. Purpose in life was related to more use of active (β = .36, *p* < .001) and support (β = .32, *p* < .001) strategies, and less use of disengaged strategies (β = −.08, *p* < .001). The associations between purpose and coping were similar across sociodemographic factors (age, sex, race, education). The association with disengaged coping differed by current distress (β_interaction_ = .23, .15, .16, respectively, for depression, anxiety, and stress, all *p*s < .001): The expected negative association was apparent for participants not in distress, whereas there was a positive association among participants experiencing depression, anxiety, or stress. Purpose in life is associated with coping strategies that can help regulate stress, which may contribute to the lower stress and better psychosocial and health outcomes among people with high purpose.

## Introduction

Purpose in life is one component of eudaimonic well-being that is related to current feelings of stress: Across a variety of populations, individuals with greater purpose in life report less subjective stress [1]. Less subjective stress may be one pathway through which purpose is related to better health outcomes [2]. That is, individuals with greater subjective stress tend to be at greater risk of poor health [3,4]. Individuals with more purpose may be less vulnerable to poor health, including lower risk of morbidity [5] and earlier mortality [6], because they experience less subjective stress that increases risk of poor health outcomes.

Individuals higher in purpose may be less reactive and/or cope better with stressors rather than experience fewer stressors in their lives. Purpose, for example, is unrelated to the number of stressors experienced in daily life, but it is associated with less reaction on days with more stressors (e.g., less increase in negative affect, fewer physical symptoms) [7]. Purpose has likewise been associated with better regulation of lab-induced stress. Although purpose was unrelated to cortisol reactivity in response to the Trier Social Stress Test, it was related to faster cortisol recovery after exposure to the stressor [8]. Individuals higher in purpose also recover faster from seeing negative images, as measured by the eyeblink startle response [9]. And, when faced with a sharp incline, individuals higher in purpose perceive it to require less effort to scale than individuals lower in purpose who face the same incline [10]. This growing literature suggests that even in the face of the same stressor, individuals higher in purpose regulate their response to it to reduce its impact. This regulation of individual stressors may culminate in feeling less subjectively stressed in general.

General coping strategies are commonly used to manage stressors, from daily hassles to stressful life events [11]. Transactional [12] and behavioral self-regulation [13] theoretical models of stress have been combined into a model of broad domains of coping strategies that are typically used to regulate stress [14,15]: active coping (approaches that reduce stressors or their effects), disengaged coping (approaches that reduce direct engagement with stressors or their effects, typically because of little confidence in one’s capacity to regulate the stressor), and support coping (approaches that emphasize social support to regulate stressors or their effects). Within Carver’s [16] taxonomy of coping strategies, the active coping domain includes active, positive reframing, planning, humor, and acceptance strategies; the disengaged coping domain includes self-distraction, denial, substance use, behavioral disengagement, venting, and self-blame strategies; and the support coping domain includes emotional support, instrumental support, and religion strategies.

There is a growing literature on the relation between purpose in life and specific coping strategies (also referred to as emotion regulation strategies). In a sample of midlife adults, for example, purpose was associated with more active strategies and fewer disengaged strategies in general [17]. In another midlife sample of adults, individuals higher in purpose were found to use more problem-focused coping and tended to use less emotion focused coping [18]. The pattern of association between purpose and coping, however, is slightly different when participants are asked about coping specifically with anxiety. In a sample of adults that ranged from 18–99 years old, purpose had the expected associations with greater engagement in active strategies, such as reframing and humor, and support strategies, such as seeking comfort from others, but it was also related to the use of more disengaged strategies, including venting and distraction, to manage (hypothetical) anxiety [19].

Individuals suffering from acute psychological distress tend to engage in less adaptive coping and more problematic strategies to cope with stress [20]. Individuals higher in purpose in life tend to be at lower risk of poor mental health [21], but purpose is not equivalent to psychological distress [22]. And, even when struggling with distress, it is still possible to feel that one’s life has purpose [23]. Such distress, however, may erode the ability to engage in more adaptive and less problematic coping strategies. Conversely, purpose in life may still support healthier coping, even when experiencing acute distress, as has been found for feelings of subjective stress [1]. Theoretical models of meaning in life suggest that purpose in life, as a component of meaning, is a motivational factor that helps individuals focus on long-term goals, and this focus may help to manage distress and psychopathology [24]. A long-term focus may provide a trajectory that helps individuals strive to attain their goals, even when facing barriers or distress. As such, the relation between purpose and coping strategies may or may not be moderated by acute distress, such as depression, anxiety, or subjective stress.

The present study examines the relation between purpose in life and coping among middle aged and older adults. We take a comprehensive approach to coping and examine both broad domains of coping (active, disengaged, support) and specific strategies within each domain. To evaluate generalizability, we test whether the associations vary by age, sex, race, or education. To test whether purpose maintains its association with more adaptive and fewer maladaptive strategies during periods of significant psychological distress, we test whether the associations vary by current elevated symptoms of depression, anxiety, or stress. We hypothesize that participants higher in purpose in will report greater use of more adaptive coping strategies (i.e., active and support strategies) and less use of disengaged strategies. We further hypothesize that these associations will generalize across sociodemographic groups and that there will be a similar pattern of associations for participants currently experiencing elevated psychological distress as those who are not.

## Materials and methods

### Participants and procedure

A survey of psychological factors and health was distributed online. Middle-aged and older adults were recruited for the survey through Dynata’s proprietary panel in March 2023. Participants recruited by Dynata through their proprietary panels were directed to a Qualtrics survey from researchers at the Florida State University. Participants were adults living in the United States; there was no limitation on where in the United States. The sample was stratified by age to have a roughly similar percentage of adults aged 40–64 and adults aged 65 and older (about 50%/50%), to have a similar percentage of male and female participants (about 50%/50%), and to be about 30% Black. The sample was stratified to provide adequate representation of populations of interest rather than sampled to be representative of the population of the United States. The analytic sample included participants who completed the purpose and coping measures. Two participants whose survey completion time was less than 5 minutes were excluded. The analytic sample included 1,998 participants. The response rate was 90.5% (n = 1,998 consented and provided valid data, n = 193 refused consent and thus did not participate, n = 15 consented but did not provide data, n = 2 consented but did not provide valid data).

### Ethics approval and consent to participate

The Florida State University Institutional Review Board approved this research (STUDY00001134). The survey was in accordance with the Declaration of Helsinki. Participants provided written informed consent before completing the survey.

### Measures

#### Purpose in life.

Purpose in life was measured with the NIH PROMIS measure of purpose and meaning [25]. Four items (e.g., “My life has purpose.”) were rated from 1 (*strongly disagree*) to 5 (*strongly agree*). The mean was taken across the items to indicate greater purpose.

#### Coping.

Participants completed the Brief COPE [16] as a measure of coping. The non-specific situation version was used. The instructions were: “We are interested in how people respond when they confront difficult or stressful events in their lives. There are lots of ways to deal with stress. The next questions ask you about what you usually do and feel when you experience stressful events. Even though you may do different things and feel different emotions, try to remember what you usually do when you are under a lot of stress. When I am confronted with a difficult or stressful event, I usually…” Fourteen coping strategies were measured with two items for each strategy. These strategies can be tested individually or combined into broader domains. The three broad domains were active coping (active, positive reframing, planning, humor, acceptance), disengaged coping (self-distraction, denial, substance use, behavioral disengagement, venting, self-blame), and support coping (emotional support, instrumental support, religion). Participants rated each item from 1 (*not at all*) to 4 (*a lot*) and the mean taken to indicate the coping strategy label (i.e., higher scores indicated more use of the coping strategy).

#### Current psychological distress.

Current symptoms of depression and anxiety were assessed using the Patient Health Questionnaire-4 [26]. Two items measured depressive symptoms within the last two weeks, and two items measured anxiety symptoms within the last two weeks. Specifically, participants were asked “Over the last two weeks, how often have you been bothered by the following problems?” “Feeling down, depressed or hopeless” and “Little interest or pleasure in doing things” (depressive symptoms) and “Feeling nervous, anxious or on edge” and “Not being able to stop or control worrying” (anxiety symptoms). Response options for each item were 0 (*not at all*), 1 (*several days*), 2 (*more than half the days*), and 3 (*nearly all the time*). The two items for depressive symptoms and the two items for anxiety symptoms were summed separately and categorized at the validated cut point (≥3) for depression and anxiety [26]. The item, “How would you rate the amount of stress in your life right now?” was used to measure perceived stress [27]. Response options for the item were 1 (*no stress*), 2 (*very little stress*), 3 (*some stress*), 4 (*moderate stress*), 5 (*a lot of stress*), and 6 (*extreme stress*). The response on this item was categorized into no to mild stress (≤4) versus severe to extreme stress (≥5) based on the response options rather than a validated cut point.

#### Covariates.

Sociodemographic factors were included as covariates. Age was measured in years. Sex was categorized as 0 = male and 1 = female. Race was categorized into two dummy-coded variables that compared 1 = Black and 1 = Otherwise identified to 0 = White. Participants reported their education on a scale that ranged from 1 = did not complete high school to 7 = professional or doctorate degree.

### Statistical approach

The relation between purpose and coping strategies was tested with linear regression. Each coping domain and specific strategy was regressed on purpose; the sociodemographic factors were included as covariates. Next, we tested whether the associations were moderated by sociodemographic factors. Moderation was tested with an interaction between purpose and age, sex, race, and education. Each model controlled for the main effects and the other sociodemographic covariates. Moderation by current psychological distress was tested with an interaction between purpose and depression, anxiety, and stress. Each model controlled for the main effects and the sociodemographic covariates. Significance was set to *p* < .01 due to the number of statistical tests.

## Results and discussion

Table 1 reports the descriptive statistics for the study variables. S1 Table reports the bivariate correlations for the study variables. Table 2 reports the results of the regression analysis. Purpose was associated with the three broad coping domains in the expected ways: Participants higher in purpose reported greater use of active and support strategies and less use of disengaged strategies to manage stress. Purpose was associated positively with all the specific strategies within the active coping domain, except for humor, which was unrelated to purpose. There was more variation within the disengaged coping domain. There was the expected negative association between purpose and denial, substance use, behavioral disengagement, and self-blame strategies, but there was a positive association with self-distraction and no relation with venting. Finally, purpose was associated positively with all three support coping strategies (emotional, instrumental, religion).

**Table 1:** Descriptive statistics for all study variables.

Variable	Mean (SD) or % (n)
Age (years)	61.28 (12.02)
Age range	40-100
Sex (female)	51.9% (1037)
Race (Black)	29.2% (584)
Race (Otherwise identified)	3.8% (75)
Education	4.00 (1.51)
Purpose in life	4.14 (.86)
Depression (yes)	17.9% (357)
Anxiety (yes)	19.3% (385)
Stress (yes)	13.1% (261)
Active Coping	2.90 (.67)
Active	3.05 (.83)
Positive reframing	2.60 (.89)
Planning	3.06 (.86)
Humor	1.88 (.87)
Acceptance	2.91 (.77)
Disengaged Coping	1.86 (.57)
Self-distraction	2.37 (.84)
Denial	1.66 (.83)
Substance use	1.42 (.77)
Behavioral disengagement	1.56 (.80)
Venting	2.18 (.81)
Self-blame	1.96 (.90)
Support Coping	2.45 (.77)
Emotional support	2.37 (.90)
Instrumental support	2.43 (.89)
Religion	2.53 (1.11)

*Note*. *N*=1,998. SD=standard deviation.

**Table 2:** Association between purpose in life and coping domains and specific coping strategies.

Coping Strategy	Full Sample	Depression		Anxiety		Stress	
		No	Yes	No	Yes	No	Yes
Active	.36**	.34**	.40**	.35**	.44**	.35**	.43**
Active	.32**	.31**	.28**	.31**	.34**	.31**	.31**
Positive reframing	.34**	.32**	.39**	.33**	.43**	.32**	.36**
Planning	.29**	.27**	.33**	.28**	.36**	.28**	.39**
Humor	.03^a,b,c^	.01	.27**	−.01	.21**	−.02	.20**
Acceptance	.20**	.18**	.26**	.20**	.28**	.19**	.29**
Disengaged	−.08** ^a,b,c^	−.08*	.33**	−.09**	.15**	−.12**	.16*
Self-distraction	.09** ^a,c^	.06	.32**	.09**	.21**	.08**	.23**
Denial	−.06* ^a,b,c^	−.03	.25**	−.05	.12	−.08**	.10
Substance use	−.09** ^a,b,c^	−.11**	.26**	−.12**	.16**	−.12**	.13
Behavioral disengagement	−.14** ^a,b,c^	−.11**	.20**	−.13**	.04	−.16**	.05
Venting	.00 ^a,b,c^	.00	.22**	−.02	.17**	−.02	.21**
Self-blame	−.24** ^a,c^	−.19**	.03	−.19**	−.15**	−.23**	−.11
Support	.32**	.32**	.43**	.31**	.41**	.31**	.45**
Emotional support	.25**	.25**	.35**	.24**	.32**	.24**	.36**
Instrumental support	.24**	.23**	.30**	.22**	.30**	.22**	.34**
Religion	.27**	.27**	.39**	.26**	.38**	.26**	.40**

*Note*. *N* = 1,998. Coefficients are standardized beta coefficients from linear regression controlling for age, sex, race, and education. ^a^ Interaction with depression; ^b^ Interaction with anxiety; ^c^ Interaction with stress.

*p < .01.

**p < .001.

None of the sociodemographic characteristics moderated the association between purpose and coping (S2 Table). As such, the association between purpose and each coping domain and strategy was similar across age, males and females, Black, Otherwise identified, and White participants, and across education.

The association between purpose and coping was, however, moderated by current psychological distress. The interaction terms are in S3 Table and the beta coefficients when the sample is split by current depression, anxiety, and stress are in Table 2. An interesting pattern of moderation emerged. The association between purpose in life and disengaged coping was moderated by depression, anxiety, and stress. There was the expected negative association between purpose and disengaged coping for participants not currently experiencing acute distress, but there was a positive association for participants currently experiencing depression, anxiety, or stress. This pattern of moderation was similar for the specific coping strategies of denial, substance use, and behavioral disengagement. Venting was also moderated by depression, anxiety, and stress, such that there was no relation with purpose for participants not currently experiencing acute distress and a positive association for participants who were. Depression and stress moderated the association with self-distraction such that there was a positive association across both groups but stronger among participants currently experiencing depression or stress. Finally, there was a negative association between purpose and self-blame among participants not currently experiencing depression or stress and a null association for participants who were. Current psychological distress did not moderate the association between purpose and either active or support coping or any of the more specific coping strategies within these domains. The one exception was humor: Purpose was associated with greater use of humor among participants currently experiencing depression, anxiety, or stress, whereas purpose and humor were unrelated among participants not experiencing these aspects of distress.

## Conclusions

This research examined how purpose in life was associated with coping strategies and whether these associations were moderated by sociodemographic factors or current psychological distress. As expected, purpose was associated with more use of active and support coping and less use of disengaged coping. The associations between purpose and coping were similar across age, sex, race, and education. Contrary to expectations, aspects of disengaged coping were moderated by current distress. Purpose generally had negative or no association with disengaged coping among participants not currently experiencing distress, but purpose had positive associations with these strategies among participants who were in acute distress.

Purpose in life reflects the feeling that one has a life that is goal oriented and has direction [28]. Integral to this theoretical definition is the ability to continue to strive for one’s goals despite obstacles to those goals that may arise [29]. As such, coping that helps to manage obstacles may be essential to maintaining goal-directed activity in pursuit of one’s purpose. This association is unlikely to be one-directional [30]. That is, having greater purpose in life may help to develop stress regulation strategies that may help maintain goal pursuit. At the same time, it is also likely that stress regulation that supports goal pursuit may help strengthen purpose because achieving goals despite barriers may increase motivation to pursue more goals. This process may lead to a virtuous cycle where individuals higher in purpose deploy beneficial stress regulation strategies in pursuit of their goals, and achievement of goals despite obstacles strengthens resolve to pursue meaningful goals.

Purpose in life has been associated consistently with better health outcomes, including lower risk of depression [21], cardiovascular disease [6], and dementia [31], and, ultimately, lower risk of premature mortality [6]. Several mechanisms have been hypothesized to be in the pathway from purpose and better health outcomes. Individuals higher in purpose, for example, are more physically active [32] and less likely to use substances [33], and such a healthier lifestyle may help support better health. Stress has been hypothesized to be a mechanism in this relation, such that individuals higher in purpose report less subjective stress, which may lower risk of chronic disease [2].

How individuals cope with stress may likewise be a mechanism in this pathway. There are at least two non-mutually exclusive ways in which coping may be a mechanism. First, coping may help reduce the impact of a stressor and lower feelings of subjective stress. That is, when faced with a stressor, individuals higher in purpose may use strategies that are particularly beneficial for helping to manage stress, such as active coping. Second, these strategies themselves might be mechanisms of better health. Individuals who use active and support strategies to manage stress, for example, tend to have better health outcomes, whereas individuals who use disengaged strategies tend to have worse outcomes [20,34]. Disengaged strategies (e.g., strategies that tend to avoid or minimize the stressor) may help regulate stress in the moment but may not be beneficial in the long-term, such that these strategies may actually increase the stress and the correlates of the stress over time [35]. The use of these strategies may thus contribute to poor health [36,37]. The present research suggests that individuals higher in purpose tend to use more active and support strategies and less disengaged strategies, a pattern associated with better health.

As expected, the association between purpose and the coping strategies was not moderated by sociodemographic factors. The lack of moderation suggests that the associations between purpose and coping were similar across age, sex, race, and education. These findings are consistent with a recent study on purpose and stress that found the negative association between purpose and subjective stress was similar across sociodemographic populations and countries [1]. Unexpectedly, the association between purpose and disengaged coping strategies was moderated by acute distress. Interestingly, this pattern was similar to recent findings on coping specifically with anxiety – purpose was associated positively with use of disengaged strategies, as well as active and support strategies, to manage hypothetical anxiety (participants were not necessarily experiencing anxiety at the moment of making the ratings [19]). It is possible that when faced with regulating negative emotions, individuals higher in purpose would use the most strategies available that might reduce the distress. The present findings are slightly different in that the associations varied for participants currently feeling acute distress (depression and stress, as well as anxiety) and were for coping in general, rather than coping specifically with hypothetical anxiety. The pattern of moderation, however, is notable. Specifically, even when experiencing acute distress, be it depression, anxiety, or stress, individuals higher in purpose in life still engaged in coping strategies that are known to help regulate stress and are associated with better health outcomes [38,39]. As such, acute distress does not erode the ability to engage in such beneficial strategies, it may just engage additional strategies for regulating stress. People higher in purpose (and who are stressed) seem to respond more, and engage more, even if some of the coping strategies are not ideal, and higher purpose seems to translate into a greater tendency to cope with the stressor.

Although this pattern was unexpected, it may be consistent with theoretical models of regulatory flexibility [40]. Regulatory flexibility includes both motivational and mechanistic components that are engaged when faced with stressful situations. The motivational component refers to the motivation to regulate the stress. The mechanistic component includes the context, coping repertoire, and feedback. Individuals who have a larger repertoire of coping strategy have more strategies to deploy depending on both the context and feedback (i.e., whether the strategies worked or not). In the context of the present findings, purpose in life may be the motivational component, such that individuals higher in purpose in life may be motivated to regulate distress to continue to make progress toward their long-term goals, since purpose is defined, in part, as striving for goal attainment regardless of barriers [41]. When such individuals face distress (context sensitivity), a variety of strategies may be deployed (repertoire), until the stress is managed (feedback). In the absence of distress or other context that needs regulation, individuals higher in purpose may not need to deploy numerous coping strategies. The option to use more strategies if needed, however, may be available.

The present research had several strengths. The strengths included the relatively large sample, that coping was measured with a relatively comprehensive measure, and the consideration of acute psychological distress in the association between purpose and coping. Future research could address some limitations of the present work. First, the coping scale measured how individuals generally cope with stress, but it did not measure the process of coping in real time. Future research would benefit from real-time measurement of coping, particularly in daily life. Second, the sample was limited to the United States and specific populations within it (e.g., middle aged and older adults). As such, the associations might not be generalizable to other populations, although there were no significant demographic moderators of the associations between purpose and coping, and the association between purpose and subjective stress tends to be similar across populations [1]. Further, the use of Dynata to recruit participants may have introduced potential sampling bias because digital platform users may include “professional survey takers” or individuals with higher technological literacy than the general population. Third, interactions are difficult to replicate [42]. Although similar across three measures of distress, the interaction needs to be replicated in an independent sample to determine its robustness. Finally, the data were cross-sectional, so it was not possible to evaluate the temporal relation between purpose and coping or test coping as a mediator between purpose and long-term health outcomes. Longitudinal assessments are needed to test potential bidirectional associations because it is possible that successful coping may reinforce one's sense of purpose, and one’s sense of purpose may contribute to coping strategy use. In addition to longitudinal data, it would be worthwhile to take an experimental approach and test whether therapies designed to experimentally increase purpose and meaning in life (e.g., meaning-centered therapy) [43] leads to measurable changes in coping strategies to provide more robust support for the current findings. If supported by experimental evidence, there are practical implications for purpose and coping. Specifically, interventions to increase purpose may help support more adaptive coping strategies that individuals can deploy in times of stress. The development of beneficial coping strategies may help individuals to reduce the likelihood of progressing to elevated distress by deploying adaptive coping.

Despite these limitations, the present research provides a comprehensive evaluation of the association between purpose in life and coping and how acute distress modifies the associations. This work contributes to the literature on purpose and coping and informs future work to better address how the process of coping supports the better outcomes associated with purpose.

## Supporting information

S1 Table Correlations among all study variables. (DOCX)

S2 Table Interaction terms between purpose and sociodemographic factors. (DOCX)

S3 Table Interaction terms between purpose and acute depression, anxiety, and stress. (DOCX)
