# Supplementary material for: Purpose in life and coping strategies: Main associations and moderation by concurrent distress
Source: PLoS One. 2026 May 21;21(5):e0347777. doi: 10.1371/journal.pone.0347777 (PMC13193536; doi:10.1371/journal.pone.0347777)
Supplement: S1 Table — (DOCX) [file pone.0347777.s001.docx]

Supplemental Table S1

*Correlations among all study variables*

|  | 1. | 2. | 3. | 4. | 5. | 6. | 7. | 8. | 9. | 10. |
| --- | --- | --- | --- | --- | --- | --- | --- | --- | --- | --- |
| 1. Age | -- |  |  |  |  |  |  |  |  |  |
| 2. Sex | -.06** | -- |  |  |  |  |  |  |  |  |
| 3. Race (Black) | -.43** | .11** | -- |  |  |  |  |  |  |  |
| 4. Race (Otherwise) | -.03 | .03 | -.13** | -- |  |  |  |  |  |  |
| 5. Education | .06** | -.16** | -.20** | .04 | -- |  |  |  |  |  |
| 6. Purpose in life | .12** | -.02 | .02 | -.05* | .01 | -- |  |  |  |  |
| 7. Depression | -.34** | .02 | .15** | -.01 | .00 | -.37** | -- |  |  |  |
| 8. Anxiety | -.34** | .06** | .17** | -.03 | -.02 | -.27** | .70** | -- |  |  |
| 9. Stress | -.22** | .08** | .09** | -.04 | -.03 | -.22** | .48** | .52** | -- |  |
| 10. Active Coping | .07** | -.02 | -.02 | -.04 | .05* | .37** | -.09** | -.03 | -.04 | -- |
| 11. Active | .12** | -.04 | -.06* | -.04 | .07** | .33** | -.14** | -.09** | -.08** | .84** |
| 12. Positive reframing | -.07** | .02 | .08** | -.04 | -.03 | .33** | -.01 | .03 | -.02 | .77** |
| 13. Planning | .11** | -.02 | -.05* | -.04 | .07** | .30** | -.10** | -.05* | -.02 | .86** |
| 14. Humor | -.27** | -.11** | .08** | -.05* | .02 | .00 | .23** | .18** | .08** | .24** |
| 15. Acceptance | .08** | .00 | -.06** | -.01 | .05* | .20** | -.04* | .01 | .01 | .74** |
| 16. Disengaged Coping | -.40** | .00 | .16** | -.04 | .01 | -.13** | .47** | .43** | .28** | .20** |
| 17. Self-distraction | -.22** | .10** | .10** | -.04 | -.02 | .07** | .15** | .18** | .14** | .34** |
| 18. Denial | -.30** | .01 | .18** | -.02 | -.04 | -.09** | .40** | .36** | .22** | .04 |
| 19. Substance use | -.39** | -.07** | .18** | -.03 | .00 | -.12** | .42** | .38** | .24** | -.01 |
| 20. Behavioral disengagement | -.37** | .00 | .16** | .01 | .01 | -.18** | .47** | .41** | .26** | -.04 |
| 21. Venting | -.18** | .06** | .07** | -.03 | .03 | -.02 | .22** | .20** | .18** | .28** |
| 22. Self-blame | -.23** | .01 | .04* | .00 | .05* | -.26** | .41** | .37** | .27** | .09** |
| 23. Support Coping | -.13** | .14** | .14** | -.05 | -.02 | .30** | .07** | .08** | .08** | .51** |
| 24. Emotional support | -.10** | .08** | .04 | -.08** | .01 | .24** | .06** | .05* | .05* | .41** |
| 25. Instrumental support | -.08** | .04 | .01 | -.03 | .03 | .22** | .05* | .03 | .06* | .47** |
| 26. Religion | -.13** | .19** | .24** | -.01 | -.08** | .26** | .06* | .08** | .07** | .35** |

Supplemental Table S1 Continued

|  | 11. | 12. | 13. | 14. | 15. | 16. | 17. | 18. | 19. | 20. |
| --- | --- | --- | --- | --- | --- | --- | --- | --- | --- | --- |
| 11. Active | -- |  |  |  |  |  |  |  |  |  |
| 12. Positive reframing | .48** | -- |  |  |  |  |  |  |  |  |
| 13. Planning | .73** | .51** | -- |  |  |  |  |  |  |  |
| 14. Humor | .11** | .32** | .14** | -- |  |  |  |  |  |  |
| 15. Acceptance | .48** | .43** | .51** | .18** | -- |  |  |  |  |  |
| 16. Disengaged Coping | .06* | .26** | .11** | .64** | .20** | -- |  |  |  |  |
| 17. Self-distraction | .22* | .33** | .26** | .31** | .28** | .59** | -- |  |  |  |
| 18. Denial | -.04 | .15** | -.04 | .35** | .04* | .74** | .29** | -- |  |  |
| 19. Substance use | -.08** | .08** | -.06** | .39** | .03 | .72** | .24** | .51** | -- |  |
| 20. Behavioral disengagement | -.15** | .08** | -.12** | .39** | .06** | .78** | .29** | .63** | .60** | -- |
| 21. Venting | .20** | .20** | .26** | .32** | .26** | .67** | .37** | .39** | .36** | .37** |
| 22. Self-blame | .00 | .07** | .10** | .32** | .12** | .72** | .32** | .45** | .42** | .52** |
| 23. Support Coping | .38** | .50** | .41** | .27** | .34** | .37** | .39** | .26** | .19** | .20** |
| 24. Emotional support | .34** | .38** | .33** | .24** | .27** | .35** | .36** | .22** | .20** | .20** |
| 25. Instrumental support | .37** | .41** | .42** | .28** | .30** | .33** | .33** | .19** | .16** | .16** |
| 26. Religion | .23** | .40** | .25** | .14** | .25** | .22** | .25** | .20** | .10** | .14** |

Supplemental Table S1 Continued

|  | 21. | 22. | 23. | 24. | 25. | 26. |
| --- | --- | --- | --- | --- | --- | --- |
| 21. Venting | -- |  |  |  |  |  |
| 22. Self-blame | .43** | -- |  |  |  |  |
| 23. Support Coping | .36** | .15** | -- |  |  |  |
| 24. Emotional support | .36** | .13** | .83** | -- |  |  |
| 25. Instrumental support | .35** | .14** | .83** | .74** | -- |  |
| 26. Religion | .18** | .09** | .74** | .32** | .32** | -- |
