# Supplementary material for: Purpose in life and coping strategies: Main associations and moderation by concurrent distress
Source: PLoS One. 2026 May 21;21(5):e0347777. doi: 10.1371/journal.pone.0347777 (PMC13193536; doi:10.1371/journal.pone.0347777)
Supplement: S2 Table — (DOCX) [file pone.0347777.s002.docx]

Supplemental Table S2

*Interaction terms between purpose and sociodemographic factors*

| Coping domain/Strategy |  | Age | |  | Sex | |  | Race (Black) | | Race (Other) | |  | Education | |
| --- | --- | --- | --- | --- | --- | --- | --- | --- | --- | --- | --- | --- | --- | --- |
|  |  | β | p |  | β | p |  | β | p | β | p |  | β | p |
| Active |  | .00 | .841 |  | .02 | .545 |  | -.01 | .791 | .00 | .900 |  | -.01 | .496 |
| Active |  | .01 | .619 |  | .04 | .264 |  | -.03 | .190 | .00 | .914 |  | -.01 | .790 |
| Positive reframing |  | .03 | .201 |  | .02 | .556 |  | .00 | .902 | .01 | .725 |  | -.01 | .754 |
| Planning |  | -.02 | .336 |  | .00 | .938 |  | .01 | .614 | .01 | .621 |  | -.02 | .412 |
| Humor |  | .00 | .884 |  | .02 | .649 |  | .00 | .958 | .02 | .336 |  | -.02 | .285 |
| Acceptance |  | -.01 | .767 |  | .01 | .798 |  | .00 | .924 | -.03 | .148 |  | -.02 | .438 |
| Disengaged |  | -.05 | .024 |  | .00 | .989 |  | .02 | .334 | .02 | .475 |  | -.02 | .471 |
| Self-distraction |  | -.02 | .315 |  | .01 | .705 |  | .02 | .404 | .01 | .533 |  | -.02 | .280 |
| Denial |  | -.03 | .177 |  | .01 | .734 |  | .02 | .393 | -.02 | .488 |  | .00 | .908 |
| Substance use |  | -.02 | .134 |  | .00 | .910 |  | .00 | .987 | -.02 | .324 |  | .01 | .595 |
| Behavioral disengagement |  | -.04 | .035 |  | -.03 | .345 |  | .02 | .450 | .02 | .277 |  | -.04 | .083 |
| Venting |  | -.05 | .021 |  | .05 | .137 |  | .05 | .088 | .05 | .021 |  | -.02 | .429 |
| Self-blame |  | -.04 | .043 |  | -.05 | .093 |  | .01 | .755 | .00 | .911 |  | .02 | .477 |
| Support |  | .00 | .960 |  | .04 | .181 |  | .00 | .856 | .01 | .563 |  | .00 | .953 |
| Emotional support |  | .02 | .500 |  | .06 | .080 |  | -.04 | .101 | .01 | .676 |  | .02 | .390 |
| Instrumental support |  | -.01 | .651 |  | .04 | .277 |  | .02 | .430 | .02 | .509 |  | .01 | .603 |
| Religion |  | -.01 | .772 |  | .01 | .686 |  | .03 | .273 | .01 | .764 |  | -.03 | .199 |

*Note*. Beta coefficients are the interaction term between purpose and each sociodemographic factor, controlling for

the main effects and the other sociodemographic factors. Race compared Black and Otherwise identified participants

to White participants.
