# Supplementary material for: Purpose in life and coping strategies: Main associations and moderation by concurrent distress
Source: PLoS One. 2026 May 21;21(5):e0347777. doi: 10.1371/journal.pone.0347777 (PMC13193536; doi:10.1371/journal.pone.0347777)
Supplement: S3 Table — (DOCX) [file pone.0347777.s003.docx]

Supplemental Table S3

*Interaction terms between purpose and acute depression, anxiety, and stress*

| Coping domain/Strategy |  | Depression | |  | Anxiety | |  | Stress | |
| --- | --- | --- | --- | --- | --- | --- | --- | --- | --- |
|  |  | β | p |  | β | p |  | β | p |
| Active |  | -.03 | .278 |  | .00 | .855 |  | .02 | .460 |
| Active |  | -.07 | .021 |  | -.02 | .483 |  | -.01 | .697 |
| Positive reframing |  | -.01 | .644 |  | .02 | .428 |  | .01 | .677 |
| Planning |  | -.02 | .449 |  | .00 | .995 |  | .03 | .199 |
| Humor |  | .15 | <.001 |  | .13 | <.001 |  | .12 | <.001 |
| Acceptance |  | .00 | .887 |  | .01 | .645 |  | .03 | .312 |
| Disengaged |  | .23 | <.001 |  | .15 | <.001 |  | .16 | <.001 |
| Self-distraction |  | .13 | <.001 |  | .06 | .022 |  | .08 | .004 |
| Denial |  | .17 | <.001 |  | .11 | <.001 |  | .12 | <.001 |
| Substance use |  | .25 | <.001 |  | .18 | <.001 |  | .14 | <.001 |
| Behavioral disengagement |  | .19 | <.001 |  | .09 | <.001 |  | .12 | <.001 |
| Venting |  | .12 | <.001 |  | .10 | <.001 |  | .12 | <.001 |
| Self-blame |  | .13 | <.001 |  | .04 | .120 |  | .07 | .006 |
| Support |  | .00 | .911 |  | .02 | .558 |  | .04 | .081 |
| Emotional support |  | .03 | .374 |  | .02 | .464 |  | .04 | .109 |
| Instrumental support |  | .00 | .898 |  | .02 | .422 |  | .04 | .094 |
| Religion |  | -.02 | .533 |  | .00 | .942 |  | .02 | .377 |

*Note*. Beta coefficients are the interaction term between purpose and acute distress,

controlling for the main effects and the sociodemographic factors.
